# Circular RNA expression profiling of granulosa cells in women of reproductive age with polycystic ovary syndrome

**DOI:** 10.1007/s00404-019-05129-5

**Published:** 2019-04-01

**Authors:** Chunren Zhang, Jianqiao Liu, Maohua Lai, Juan Li, Jiahui Zhan, Qidan Wen, Hongxia Ma

**Affiliations:** 1 Department of Traditional Chinese Medicine, The First Affiliated Hospital of Guangzhou Medical University, No.151 Yanjiang Road, Yuexiu District, Guangzhou, 510120 Guangdong China; 2 Department of Reproductive Medicine, Third Affiliated Hospital of Guangzhou Medical University, Guangzhou, 510150 China

**Keywords:** Circular RNA, Expression profiling, PCOS, Reproductive age

## Abstract

**Purpose:**

This study aimed to explore the expression profiles of circRNA in granulosa cells of women of reproductive age with polycystic ovary syndrome (PCOS).

**Methods:**

Total RNA was isolated from granulosa cells of 15 women with PCOS and 15 body mass index- and age-matched healthy women (control). RNA sequencing was conducted on ribosomal-depleted RNA for circRNA expression profiling. The differential expression of circRNA between women with PCOS and controls was compared and visualized using hierarchical clustering heat maps and Volcano plots. Gene ontology (GO) and Kyoto Encyclopedia of Genes and Genomes pathway enrichment analyses were performed to determine the role of the differential expression of circRNAs. The expression rates of circRNAs were confirmed using quantitative real-time PCR (qRT-PCR) using divergent primers.

**Results:**

A total of 4258 and 7395 candidate circRNAs were predicted in PCOS and controls, respectively, based on the RNA-sequencing data. Differences were noted in the expression patterns of circRNA between the two groups. Analysis of the expression profiles revealed that four circRNAs were upregulated, whereas 23 were downregulated in the women with PCOS. GO analysis suggested that the 27 differentially expressed circRNAs were mainly distributed in biological process pathways, particularly in pathways involving inflammation, proliferation, and the vascular endothelial growth factor-related signaling pathway. Six circRNAs were identified in PCOS-affected women using divergent primers. qRT-PCR confirmed that hsa_circ_0001577 was significantly upregulated and hsa_circ_0020093 was downregulated in the women with PCOS.

**Conclusions:**

Several circRNAs were differentially expressed in women of reproductive age with PCOS, suggesting the involvement of these circRNAs in the development of PCOS and the potential clinical implications of their use as PCOS biomarkers.

**Electronic supplementary material:**

The online version of this article (10.1007/s00404-019-05129-5) contains supplementary material, which is available to authorized users.

## Introduction

Polycystic ovary syndrome (PCOS) is an endocrinopathy that is characterized by oligomenorrhoea/amenorrhoea, hyperandrogenism, and polycystic ovaries [1]. It is the most common cause of anovulatory infertility, affecting 5–20% of women of reproductive age worldwide [2, 3]. The etiology of PCOS remains unclear. It is estimated that the annual impact of PCOS on the US health budget is approximately $4.36 billion per year [4]. There is, therefore, an urgent need of investigation into molecules involved in the etiology of PCOS and the subsequent development of diagnostic biomarkers.

Generally, PCOS is diagnosed using the criteria developed by the European Society of Human Reproduction and Embryology/American Society for Reproductive Medicine (ESHRE/ASRM, Rotterdam). Two of three phenotype features must be present: hyperandrogenism and/or hyperandrogenemia; oligoovulation; and polycystic ovarian morphology [5]. It has been suggested that clinical features including insulin resistance, an increased risk of pregnancy-related complications such as anovulatory infertility, miscarriage [6], type 2 diabetes mellitus, endometrial cancer [7], and cerebrovascular and cardiovascular pathologies are also associated with PCOS in some women [8, 9]. With the development of high-throughput, next-generation sequencing technologies, changes in gene expression that are related to disease initiation and progression are increasingly easy to detect. Therefore, the use of high-throughput technologies for investigating novel biomarkers would be of significant benefit in the diagnosis of PCOS.

Circular RNAs (circRNAs) are a novel class of endogenous RNAs that are generated from precursor mRNA (pre-mRNA) using head-to-tail backsplicing. They function as miRNA sponges. Unlike traditional linear RNAs, circRNAs form covalent closed-loop structures that do not have typical 5ʹ caps and 3ʹ polyA tails [10]. Although the functions of several circRNAs have not been fully investigated, there is some evidence demonstrating that circRNAs are differentially expressed in a range of serious diseases, including cancer [11], Alzheimer’s disease [12], heart dysfunctions, [13] and endocrine dysfunctions such as type-2 diabetes mellitus [14]. These observations of aberrant expression of circRNA in these diseases suggest the role of circRNA as diagnostic and prognostic biomarkers.

In the field of PCOS, previous studies have demonstrated that endogenous microRNAs (miRNAs), which are differentially expressed in cumulus granulosa cells (GCs), serum, and follicular fluid, are associated with PCOS [15–17]. For long noncoding RNAs (lncRNAs), the aberrant expression of a variety of lncRNAs has also been observed in cumulus cells isolated from patients with PCOS. However, little is known about the circRNA profiles of patients with PCOS, especially of women of reproductive age. In this study, we recruited women with PCOS and conducted RNA sequencing (RNA-seq) using RNA isolated from GCs to explore the expression profiles of circRNA in PCOS.

## Materials and methods

### Patient recruitment and study design

The study was approved by the Institute Research Medical Ethics Committee of the Third Affiliated Hospital of the Guangzhou Medical University. All participants provided informed consent before recruitment. A total of 30 patients were enrolled in the study consecutively; of these 15 were women with PCOS and the remaining 15 were body mass index (BMI)- and age-matched non-PCOS controls who underwent in vitro fertilization (IVF) intracytoplasmic sperm injection (ICSI) treatment. Patients exhibiting two of the following three conditions, oligoovulation or anovulation, hyperandrogenism, or polycystic ovaries, were diagnosed with PCOS as specified by the Rotterdam 2003 criteria [18]. Patients with congenital adrenocortical hyperplasia, Cushing’s syndrome, ovarian or adrenal tumors, hypothyroidism, or hyperprolactinemia were excluded from this study. The clinical characteristics of the patients and controls are detailed in Table 1.

**Table 1:** Baseline characteristics of participants

Items	Controls (*n* = 15)	PCOS (*n* = 15)	*P* value
Age (years)	29.73 ± 3.39	29.20 ± 2.42	0.624
BMI (kg/m^2^)	19.84 ± 1.00	20.82 ± 1.68	0.067
estrogen (pg/mL)	141.08 ± 32.25	172.86 ± 77.36	0.153
Prolactin (ng/mL)	0.85 ± 0.28	0.89 ± 0.33	0.693
LH (mIU/mL)	3.46 ± 1.22	12.28 ± 5.12	0.000*
FSH (mIU/mL)	6.20 ± 1.15	4.71 ± 0.99	0.001*
Fasting insulin (μU/mL)	4.600 ± 1.200	10.55 ± 2.051	0.2054
Total cholesterol (mg/dL)	4.354 ± 0.2188	4.600 ± 0.1858	0.3998
Triglycerides (mg/dL)	4.354 ± 0.2188	1.471 ± 0.3326	0.000*
Anti-Müllerian Hormone (ng/mL)	3.85 ± 1.82	11.88 ± 1.37	0.000*
Testosterone (ng/mL)	0.92 ± 0.21	2.05 ± 0.52	0.000*

Data were analyzed by *t* test

*P < 0.05

### Collection of follicular fluid (FF) and isolation of GCs

FF was collected from patients and controls and centrifuged at 2500 g for 10 min. After removing the supernatant, samples were resuspended in phosphate-buffered saline (PBS). To isolate GCs from FF, PBS-resuspended samples were run through a 50% Percoll gradient (Sigma, St. Louis, USA), followed by centrifugation at 1000 g for 20 min at 4 °C as previously described [19, 20], with minor modifications. GCs were collected from the second layer of the liquid. The second, milky white layer was carefully collected using a pipette and then resuspended in PBS. GCs were further purified by straining and were then stored at −80 °C in the TRIzol reagent (Thermo-Fisher Scientific, USA) to be used for RNA isolation.

### RNA isolation and RNA-seq

Total RNA from GCs was isolated using the TRIzol reagent following a standard protocol. The quality of the RNA obtained, including the integrity and size distribution, was confirmed using the Agilent 2100 Bioanalyzer pico-RNA chip (Aglient, CA, USA). Before the construction of an RNA-seq library, rRNA was removed from the total RNA samples using the RiboMinus Eukaryote Kit (Qiagen, Hilden, Germen). The NEB Next Ultra Directional RNA Library Prep Kit for Illumina (NEB, MA, USA) was used according to the manufacturer’s instructions to create an RNA library for RNA-seq. The resulting RNA-seq library was quantified using an Agilent 2100 Bioanalyzer and was run on the HiSeq 2000 platform (Illumina, CA, USA) for RNA sequencing.

### CircRNA prediction and differentially expressed circRNA analyses

Clean RNA-seq data were aligned to the human GRCh37/hg19 reference genome using the Burrows–Wheeler Alignment Tool 0.7.13. Candidate circRNAs were predicted and annotated using the CIRI software. The circRNAs, which were differentially expressed between the PCOS samples and controls, were identified using the reads per kilobase per million mapped reads algorithm, and the circRNA reads were normalized to the total number of reads. *p* values obtained by a previously reported model [21] were adjusted using the Benjamini and Hochberg’s approach for controlling the false discovery rate. The circRNAs meeting the criterion of log_2_ (PCOS/control) > 1 and adjusted *p* value < 0.05 were considered to be differentially expressed circRNAs.

For the analysis of circRNA expression, the differentially expressed circRNAs were analyzed using the R (version 1.0.8) heatmap package (https://cran.r-project.org/web/packages/pheatmap/) for hierarchical clustering analysis and were distinguished using the volcano plot method. Gene ontology (GO) analysis was performed to explore the functional roles of differentially expressed circRNAs. Biological pathways in which the differentially expressed circRNAs were enriched were identified using the Kyoto Encyclopedia of Genes and Genomes (KEGG) (http://www.genome.jp/kegg/) database (adjusted *p* value < 0.05; gene count ≥ 2).

### Identification and quantification of circRNA

To confirm the backsplicing sites of the circRNAs, and to quantify the differences in expression between patients with PCOS and controls, the RNA was reverse transcribed to cDNA using PrimeScript RT Master Mix (Takara, Dalian, China) and random primers. Candidate circRNAs were amplified using divergent primers designed for backsplicing sites (Supplementary Table S1). Genomic DNA samples were isolated from GCs using the QIAamp DNA Mini Kit (Qiagen, Hilden, Germany), and cDNA amplified by convergent primers were used as controls. The products produced by amplification using the convergent and divergent primers were purified using Sanger sequencing (Sangon Biotech, Shanghai, China) to verify the backsplicing site sequence.

For the quantification of circRNA expression, real-time PCR analyses were performed using SYBR Premix Ex Taq II with divergent primers (Takara, Dalian, China). The relative expression levels of circRNAs were calculated using the 2^−ΔΔCt^ method.

### Statistical analyses

Results are expressed as mean ± SD. Comparisons between two groups were performed using the Student’s *t* test. Significant differences among two or more groups were estimated using the one-way analysis of variance using the SPSS 19.0 (SPSS Inc., IL, USA). A *p* value of < 0.05 was considered to be statistically significant.

## Results

### Overview of circRNA expression in patients with PCOS

To investigate the expression profiles of circRNA in patients with PCOS, a total of 30 women (15 with PCOS and 15 BMI- and age-matched healthy women) were recruited (Table 1) and subjected to RNA-seq analysis. RNA-seq revealed that the expression patterns of circRNA in patients with PCOS were different from those in the controls. A total of 42,244 and 85,210 reads were generated for patients with PCOS and controls, respectively. After redundant data were removed, 4258 and 7395 candidate circRNAs were identified in patients with PCOS and controls, and of these, 2899 (68.08%) and 4415 (59.70%) were included in circBase, respectively. Of these circRNAs, exon-derived circRNAs were present in similar proportions in patients with PCOS and controls, accounting for 91.10% and 89.20%, along with intron circRNAs, accounting for 8.57% and 10.66%, respectively. In addition, there were 14 and 11 circRNAs that originated from intergenic regions in patients with PCOS and controls, respectively (Table 2).

**Table 2:** Summary information of candidate circRNAs

Sample	Controls	PCOS
Number of circular junction reads	42,428	85,210
Number of circRNA species	4258	7395
Number of circRNA species reported in circBase	2899 (68.08%)	4415 (59.70%)
Number of circRNA species originated from exon regions	3879 (91.10%)	6596 (89.20%)
Number of circRNA species originated from intron regions	365 (8.57%)	788 (10.66%)
Number of circRNA species originated from intergenic regions	14 (0.33%)	11 (0.15%)

### Analysis of differentially expressed circRNAs

Differential expression analysis showed that compared with the controls, 27 differentially expressed circRNAs were found in patients with PCOS, of which 4 were upregulated and 23 were downregulated. The number of downregulated circRNAs in the patients with PCOS was higher than that of upregulated circRNAs (Table 3).

**Table 3:** Differentially expressed circRNAs between PCOS patients and controls

circRNA	Chromosome	Gene symbol	log_2_ ratio (P/N)	*P* value	Up/down regulation
NA	chrY:13688616|13851691	N/A	−2.675	7.89E−72	Down
hsa_circ_0020093	chr10:116879949|116889297	ATRNL1	−1.242	2.50E−18	Down
hsa_circ_0000284	chr11:33307959|33309057	HIPK3	−1.135	4.28E−12	Down
hsa_circ_0001922	chrX:53672263|53681075	HUWE1	−2.17	4.28E−11	Down
hsa_circ_0000839	chr18:21644104|21649235	TTC39C	−1.896	1.78E−10	Down
hsa_circ_0004032	chr6:16658007|16753578	ATXN1	−2.228	6.15E−09	Down
hsa_circ_0050277	chr19:20828490|20829211	CTC-513N18.7	−2.509	1.77E−08	Down
hsa_circ_0002483	chr8:141874411|141900868	PTK2(FAK)	−1.379	1.77E−08	Down
hsa_circ_0002484	chr11:130130751|130131824	ZBTB44	−3.269	4.59E−08	Down
hsa_circ_0001414	chr4:56277781|56284152	TMEM165	−1.939	1.20E−07	Down
hsa_circ_0002290	chr11:77396150|77404656	RSF1	−3.006	1.62E−07	Down
hsa_circ_0070039	chr4:77055328|77065626	NUP54	−2.135	2.46E−07	Down
hsa_circ_0005782	chr1:21097423|21100103	HP1BP3	−1.481	4.14E−07	Down
hsa_circ_0006595	chr1:44386076|44386600	ST3GAL3	−3.076	7.17E−07	Down
hsa_circ_0004711	chr13:42385361|42393522	VWA8	−3.076	7.17E−07	Down
hsa_circ_0001103	chr2:224862832|224866639	SERPINE2	−1.257	9.94E−07	Down
hsa_circ_0005777	chr5:73136305|73136585	ARHGEF28	−1.038	1.79E−06	Down
hsa_circ_0009061	chr1:23356962|23377013	KDM1A	−1.315	3.93E−06	Down
hsa_circ_0018281	chr10:46158990|46159290	ZFAND4	−2.932	4.32E−06	Down
hsa_circ_0008012	chr4:103446669|103459113	NFKB1	−2.176	4.43E−06	Down
hsa_circ_0009022	chr18:9583115|9595100	PPP4R1	−2.121	9.41E−06	Down
hsa_circ_0123212	chr3:196118684|196134264	UBXN7	−2.121	9.41E−06	Down
hsa_circ_0005600	chr17:57430576|57430887	YPEL2	−1.066	9.47E−06	Down
hsa_circ_0024604	chr11:120276827|120278532	ARHGEF12	1.502	2.87E−42	Up
hsa_circ_0137905	chr9:126214555|126217058	DENND1A	1.296	1.78E−06	Up
hsa_circ_0005925	chr7:99952766|99953427	PILRB	2.316	6.53E−06	Up
hsa_circ_0001577	chr6:13632602|13644961	RANBP9	3.956	1.30174E−07	Up

*NA* not available, *Chromosome* chromosome location of circRNA, *log*_*2*_*Ratio(P/N)* log_2_ ratio(PCOS/control)

**Fig. 1:**
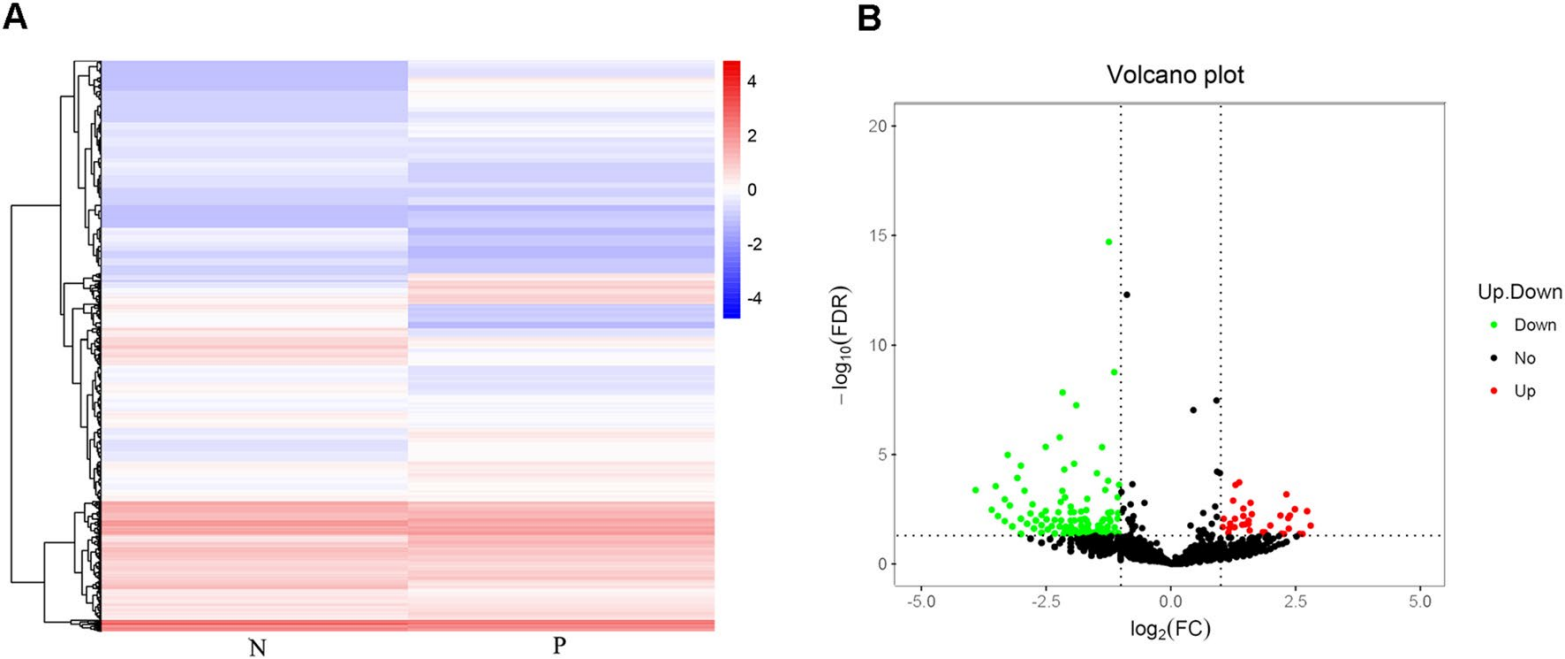
CircRNA expression profiles in GCs of patients with PCOS and controls. **a** Hierarchical clustering analysis of differentially expressed circRNAs. **b** Volcano plot showing the upregulated (in red) and downregulated (in green) circRNAs in patients with PCOS

**Fig. 2:**
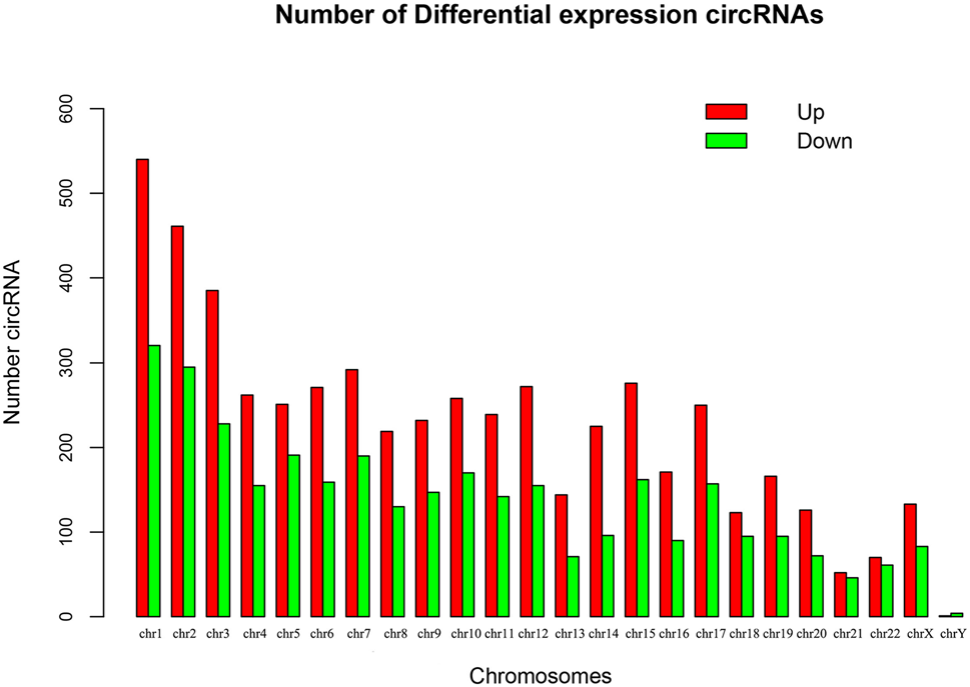
Chromosomal distribution of differentially expressed circRNAs in patients with PCOS patients

Using a hierarchical clustering heatmap to visualize the profiles of circRNAs between patients with PCOS and controls revealed differences in the expression pattern of circRNAs between the two groups (Fig. 1a). Volcano plots additionally indicated that the expression of some circRNAs was upregulated whereas that of others was downregulated (Fig. 1b). Analyzing the genomic distribution of the differentially expressed circRNAs revealed that these circRNAs tended to occur in chromosomes 1, 2, and 3 (Fig. 2).

**Fig. 3:**
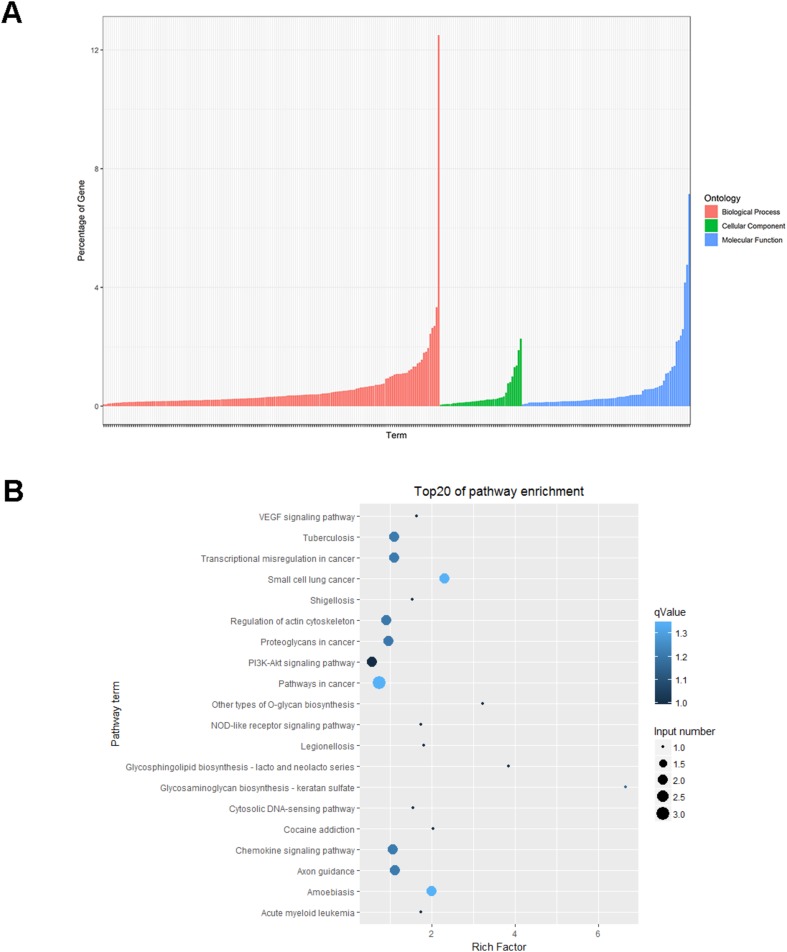
GO and KEGG analyses of differentially expressed circRNAs. **a** GO analysis **b** the top 20, most enriched KEGG pathways generated using differentially expressed circRNAs in patients with PCOS

We used GO and KEGG pathway enrichment analyses to investigate the potential functions of differentially expressed circRNAs. Differentially expressed circRNA had GO annotations to biological processes, cellular components, and molecular functions, with the most annotations to biological processes (Fig. 3). KEGG pathway enrichment analysis showed that pathways involved in cancer, the regulation of the actin cytoskeleton, the PI3 K–Akt signaling pathway, and the chemokine signaling pathway were particularly enriched with differentially expressed circRNAs.

### Identification and characterization of differentially expressed circRNAs in patients with PCOS

**Fig. 4:**
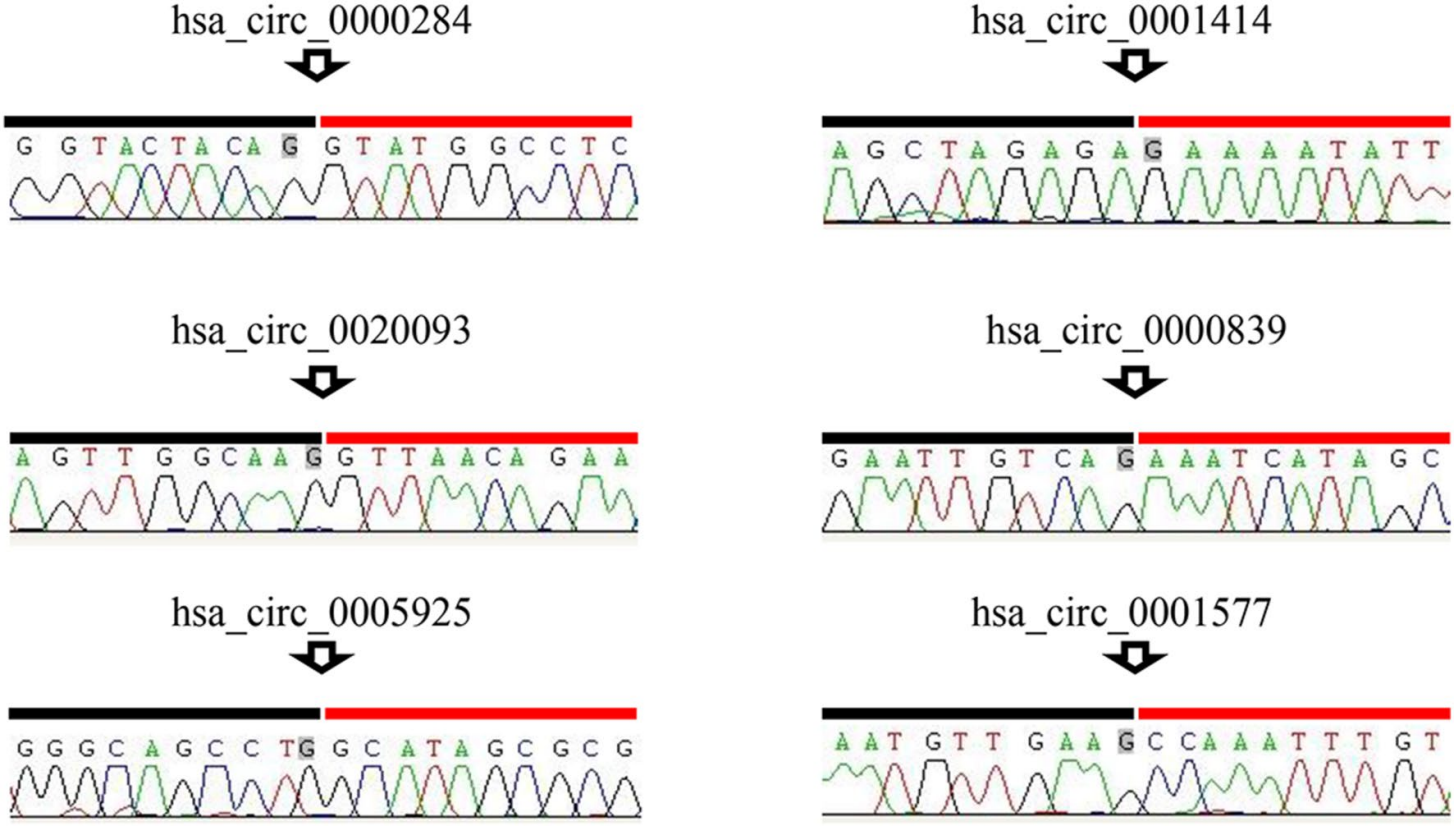
Identification of backsplicing sites of circRNAs using Sanger sequencing

To verify the backsplicing of circRNAs, two upregulated and four downregulated circRNAs were selected for analysis. cDNA and gDNA from patients with PCOS were amplified using convergent primers, designed for identifying linear RNA, and divergent primers, designed for identifying circRNA, respectively. The length of the amplified products was observed to be the same in all the convergent primers, amplified cDNA and gDNA samples of each circRNA, suggesting that linear sequences exist in both the genome and the transcript (Fig. 4). However, when circRNA-specific divergent primers were used for amplification, the expected bands only appeared in the cDNA samples, suggesting that these circRNAs were derived from the transcript and not from the genome. Linear GAPDH were also amplified as negative and positive controls. GAPDH-divergent primers were used to amplify regions that did not encode circRNAs. As expected, target bands were not observed in cDNA and gDNA samples; however, target bands were observed in those amplified using the convergent GAPDH primers. Subsequently, Sanger sequencing confirmed that the product amplified using each circRNA-specific divergent primer contained predicted backsplicing sites (Fig. 4).

**Fig. 5:**
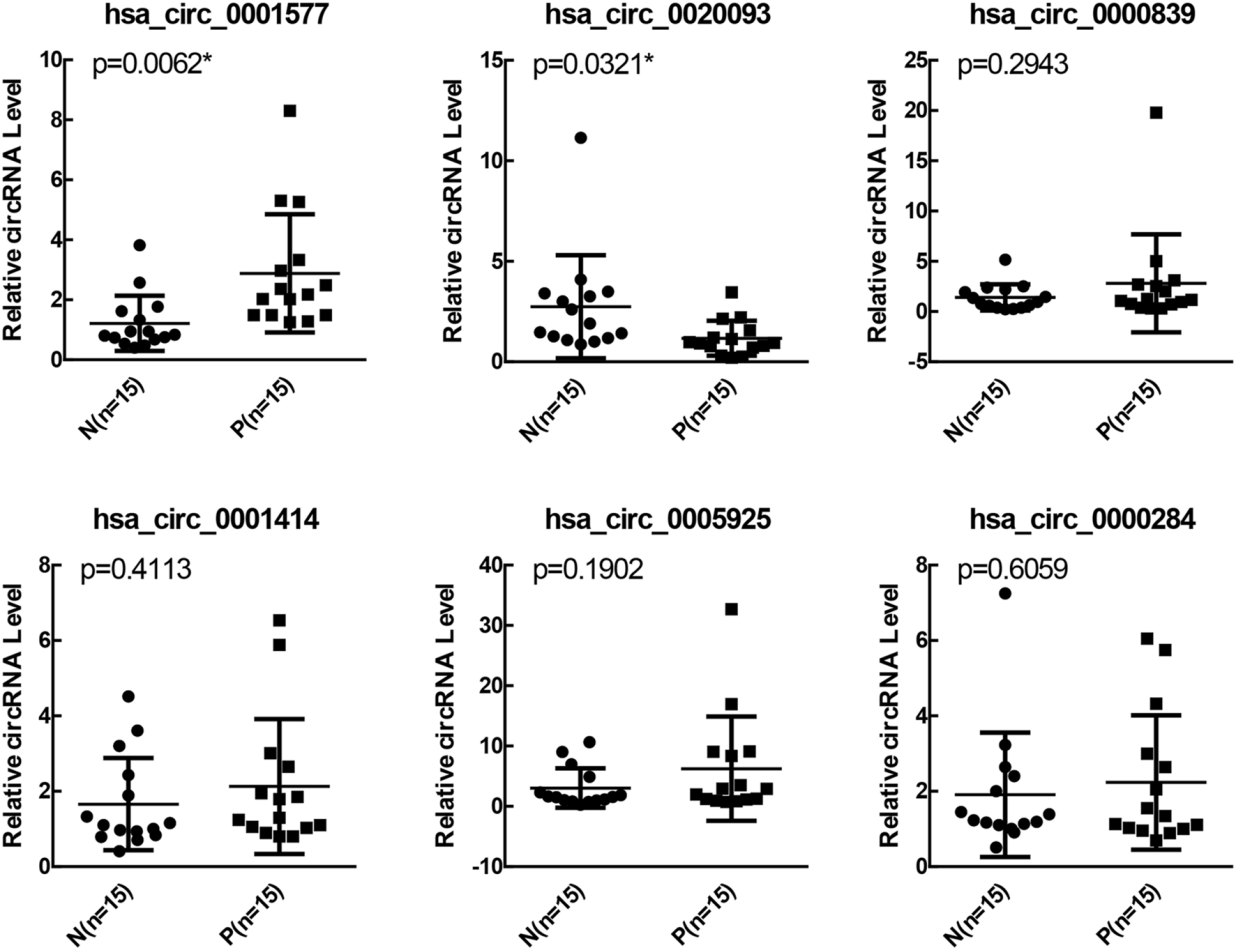
Comparison of the expression of circRNAs between patients with PCOS and controls using qRT-PCR

To verify the differential expression of circRNAs in patients with PCOS, 15 paired patients with PCOS and controls were recruited for the quantification of the expression of 2 upregulated (hsa_circ_0001577 and hsa_circ_0005925) and 4 downregulated circRNAs (hsa_circ_0020093, hsa_circ_0000839, hsa_circ_0001414, and hsa_circ_0000284). qRT-PCR confirmed that compared with the controls, hsa_circ_0001577 was significantly upregulated (*P* = 0.0062) and hsa_circ_0020093 was downregulated (*P* = 0.0321) in patients with PCOS (Fig. 5).

## Discussion

Circular RNAs are backspliced endogenous RNAs that can potentially be used as biomarkers for disease diagnosis and prognosis. However, the expression of these circRNAs has not previously been elucidated in patients with PCOS. In this study, we aimed to investigate the circRNA expression profiles of patients with PCOS. We isolated total RNA from the GCs of women of reproductive age with PCOS and from those of BMI- and age-matched controls and performed RNA-seq. Several circRNAs were found to be differentially expressed in women with PCOS, and the differentially expressed circRNAs were found to be highly clustered in pathways listed in GO biological processes and in cancer-, inflammation- and endocrine-related pathways. We also conducted qPCR assays and confirmed that hsa_circ_0001577 was upregulated and hsa_circ_0020093 was downregulated in patients with PCOS. Our findings extend knowledge about the expression profiling of a new type of endogenous RNA, which has been implicated in PCOS.

It has been reported that PCOS is the leading cause of anovulatory infertility and affects > 80% of women with anovulatory infertility [18, 22]. PCOS is often closely associated with recurrent spontaneous abortions in patients with infertility [23]. Thus, it is important that we focus on this population of women of reproductive age, who may suffer from poor outcomes because of the complications of PCOS. We, therefore, selected this subset of population for investigating circRNA expression.

Although the underlying molecular mechanism is uncertain, GCs have been demonstrated to play an important role in the pathogenesis of PCOS. It is thought that aberrant hormonal responses to GC are one of the contributors to the development of PCOS [24]. The elevated levels of luteinizing hormone (LH) and insulin resistance are typical symptoms exhibited by women with anovulation and PCOS; moreover, the dysfunction of LH and insulin interactions can affect the terminal differentiation of GCs [4, 25]. The activation of LH/chorionic gonadotrophin receptor, an important effector of ovulation in GCs of patients with PCOS, was also reported in a previous study [26], which highlighted the importance of GCs and hormones present in them. However, RNA information from the GCs of women with PCOS is currently limited.

With the development of high-throughput, next-generation sequencing, studies applying this technique for assessing PCOS demonstrate that a wide range of transcripts are differentially expressed in PCOS. Lerner et al. showed that cholesterol biosynthesis and metabolism-related genes such as hydroxy-3-methylglutaryl and CoA synthases 1 and 2 were downregulated in GCs of women with PCOS [27]. Xu et al. demonstrated miRNA expression in the cumulus GCs in patients with PCOS and suggested a pivotal role of the Notch signaling pathway in the pathophysiology of PCOS [15]. A recent study on lncRNA sequencing in cumulus cells demonstrated that 623 lncRNAs23 were differentially expressed in patients with PCOS [28]. However, till date, no study has examined circRNA expression in patients with PCOS. Our study revealed four upregulated and 23 downregulated circRNAs, suggesting that in addition to lncRNAs, miRNAs and mRNAs, circRNAs are also important in PCOS. The present study extends our knowledge regarding circRNA expression in GCs of patients with PCOS.

The circRNAs that were differentially expressed in PCOS patients were mainly centered in inflammation- and endocrine-related pathways, including chemokines, PI3 K–Akt, and vascular endothelial growth factor (VEGF) signaling pathways. It has been suggested that the apoptotic rates of GCs is low and the proliferation rate is high in patients with PCOS, indicating that cell proliferation is an important event in PCOS [29]. PI3 K–Akt is an important pathway in regulating cell proliferation and survival in several cancers [30]. This pathway is also involved in the pathology of PCOS [31, 32] and has an effect on ovarian GCs [33] as well as follicular growth [34]. These results imply that the differential expression of circRNAs involved in the pathway may be associated with GC proliferation.

It has been recognized that the VEGF signaling pathway regulates the angiogenesis, which is a prominent feature of PCOS [35]. The contribution of VEGF to follicular development was also observed in Rhesus monkeys [36]. In PCOS, the expression of VEGF was found to be induced in luteinized GCs and theca lutein cells [37, 38]. Consistent with these studies, our study also demonstrated the presence of highly enriched up- and downregulated circRNAs in the VEGF signaling pathway, suggesting a possible relationship between circRNAs and the pathway.

We also identified a chemokine pathway enriched with differentially expressed circRNAs. Chemokines are regulatory factors in the immune system. Emerging evidence indicates that inflammation-related cytokines such as chemokine (C–C motif) ligand 3, IL-18, and C-reactive protein are elevated in women with PCOS, suggesting that low-grade inflammation manifests in PCOS [39–41]. In particular, a wide range of inflammation-related genes are found to be differentially expressed in the GCs of women with PCOS [42]. We thus propose that the differentially expressed circRNAs observed in the current study may be linked to inflammatory events in PCOS.

In conclusion, although the exact function of the differentially expressed circRNAs is currently unknown, the present findings may provide information regarding the molecular mechanisms of PCOS with respect to the differential regulation of circRNA. However, these findings are based on early stage clinical experiments. In the future, we will focus on the identification of relevant circRNA biomarkers and the functional analyses of these differentially expressed circRNAs.

## Electronic supplementary material

Below is the link to the electronic supplementary material.

Supplementary material 1 (docx 13 kb)
