# Supplementary material for: Circular RNA expression profiling of granulosa cells in women of reproductive age with polycystic ovary syndrome
Source: Arch Gynecol Obstet. 2019 Apr 1;300(2):431–40. doi: 10.1007/s00404-019-05129-5 (PMC6592967; doi:10.1007/s00404-019-05129-5)
Supplement: Supplementary file 1 — Supplementary material 1 (docx 13 kb) [file 404_2019_5129_MOESM1_ESM.docx]

Supplementary table S1. Primers used in the study.

| Primer name | Sequence (-5’ to 3’-) |
| --- | --- |
| hsa_circ_0001577-CF | ACTTTGGACGAGAGCTGCAA |
| hsa_circ_0001577-CR | ACACGAAAGGATGTTGCCCA |
| hsa_circ_0005925-CF | GAGCTACCCAGAGGCTGTTG |
| hsa_circ_0005925-CR | TCCCCTTGAGATCAGGAATGA |
| hsa_circ_0000284-CF | GGGTCGGCCAGTCATGTATC |
| hsa_circ_0000284-CR | ACTGCTTGGCTCTACTTTGAGT |
| hsa_circ_0020093-CF | AATTGCGGCAGTCCAGATCA |
| hsa_circ_0020093-CR | TGGATAGCCTTCAATGAGCCA |
| hsa_circ_0001414-CF | CAGAATGGGGTGATCGCTCT |
| hsa_circ_0001414-CR | TGATATGGCAGCGACAAATGC |
| hsa_circ_0000839-CF | CCCTCTATGGTTGATCGGCT |
| hsa_circ_0000839-CR | ACTGACAAAGCTGGCTCCAAA |
